# Prediction of HER2‐Low Breast Cancer via Multimodal Ultrasound Imaging

**DOI:** 10.1002/cam4.71156

**Published:** 2025-09-02

**Authors:** Feihang Dai, Baoliang Guo, Xin Ai, Dandan Liu, Longbin Dai, Chengzhi Zhang, Xiaoping Leng, Tong Wu

**Affiliations:** ^1^ Department of Ultrasound The Second Affiliated Hospital of Harbin Medical University Harbin People's Republic of China; ^2^ Department of General Surgery The Second Affiliated Hospital of Harbin Medical University Harbin People's Republic of China; ^3^ Department of Radiology The First Affiliated Hospital of Fujian Medical University Fuzhou People's Republic of China

**Keywords:** breast cancer, contrast‐enhanced ultrasound, HER‐2 low expression, multimodal ultrasound

## Abstract

**Purpose:**

Human epidermal growth factor receptor 2 (HER2) is a key biomarker for clinical management and prognostic evaluation of breast cancer patients. This study was aimed at assisting the preoperative and non‐invasive prediction of HER2‐low breast cancer using multimodal ultrasound imaging and clinicopathological indicators, providing valuable imaging information for clinical precision diagnosis and personalized treatment strategies, especially in the application of antibody‐drug conjugates such as T‐DXd.

**Materials and Methods:**

This retrospective study included 147 pathologically confirmed breast cancer patients from two institutions: 101 in the training set and 46 in the external validation set. All patients underwent multimodal ultrasound (grayscale, color Doppler, elastography, and contrast‐enhanced imaging) and had complete clinicopathological data. Patients were categorized as HER2‐negative, HER2‐low, or HER2‐positive based on immunohistochemistry. Logistic regression was used to construct predictive models.

**Results:**

Compared with the HER2‐negative group, low Ki‐67, PR positivity, longer rise time (RT), and lower Emax values were independent predictors of HER2‐low status (*p* < 0.05), yielding an AUC of 0.876, sensitivity 0.833, and specificity 0.781. Compared with HER2‐positive cancers, HER2‐low cases showed low Ki‐67, ER/PR positivity, low Emax, and a DVPC pattern characterized by an initial increase followed by a subsequent decline as independent predictors (*p* < 0.05), with an AUC of 0.929, sensitivity 0.905, and specificity 0.856. External validation confirmed robust model performance (AUC = 0.925 and 0.918 for HER2‐low vs. negative and positive, respectively).

**Conclusion:**

A model integrating multimodal ultrasound and clinicopathological factors effectively predicts HER2‐low breast cancer, offering valuable imaging‐based support for clinical decision‐making.

**Abbreviations**
2DUS: 2D ultrasound
ADCs: antibody–drug conjugates
AUC: area under the curve
CDFI: color Doppler flow imaging
CEUS: contrast‐enhanced ultrasound
DVPC: dynamic vascular pattern curve
Emax: maximum value of the Young's modulus
Emean: mean value of the Young's modulus
Emin: minimum value of the Young's modulus
ER: estrogen receptor
Eratio: stiffness ratio of the lesion and surrounding normal tissue
FISH: fluorescence in situ hybridization
HER‐2: human epidermal growth factor receptor 2
HR: hormone receptor
IHC: immunohistochemistry
Imax: maximum intensity
ISH: in situ hybridization
Ki‐67: nuclear proliferation antigen
MRI: magnetic resonance imaging
mTT: mean transit time
NPV: negative predictive value
pCR: pathological complete response
PPV: sensitivity, positive predictive value
PR: progesterone receptor
RT: the rise time
SWE: shear wave elastography
T‐DXd: trastuzumab deruxtecan
TIC: time–intensity curve
TTP: time to peak

## 1 Introduction

Breast cancer is the most common malignancy among women globally, and its incidence and mortality rates are increasing annually. Moreover, there is a trend toward increasing diagnoses in younger patients [1]. Breast cancer is also a highly heterogeneous malignancy, and molecular‐level differences affect its biological behavior, pathology, clinical findings, imaging features, treatment, and prognosis [2]. Human epidermal growth factor receptor 2 (HER‐2) is a member of the human epidermal growth factor receptor tyrosine kinase family, which regulates cell growth and differentiation signaling pathways. It plays an important role in the biological behavior and pathogenesis of breast cancer and is a critical target for treatment and prognosis evaluation [3]. In clinical practice, the HER‐2 expression level of breast cancer is typically classified as positive or negative on the basis of immunohistochemistry (IHC) and in situ hybridization (ISH) methods. HER2‐positive breast cancer patients can benefit significantly from targeted therapies, such as trastuzumab and lapatinib [4]. Related research indicates that approximately15% to 20% of HER‐2 molecules exhibit abnormal amplification or overexpression in breast cancers.

With advanced research on molecular biology, the development of precision medicine, and the emergence of new antibody–drug conjugates (ADCs), HER2‐low breast cancer (characterized by IHC scores of 1+ or 2+ with negative fluorescence in situ hybridization) has been recognized to possess unique molecular features, holding promise as a new treatment subtype. Epidemiological data from multicenter studies have shown that the HER2‐low subtype accounts for approximately 42.8% to 59% of breast cancer cases [5]. Previous scholars have performed related studies on HER2‐low breast cancer. Zhang et al. analyzed genomic data from 523 Chinese female breast cancer patients and reported that mutations in PIK3CA and genes associated with the PI3K‐Akt signaling pathway were more common in HER2‐low breast cancer patients, whereas mutations in TP53 were more common in HER2‐negative cancer patients [6]. Zhu et al. [7] reported that compared with HER2‐negative cancers, HER2‐low cancers tended to have a higher pathological stage and grade, a higher incidence of ductal carcinoma, a higher positivity rate for estrogen receptor (ER) and progesterone receptor (PR), and a lower Ki‐67 (nuclear proliferation antigen) index. Denkert et al. [8] reported that among hormone receptor (HR)‐positive breast cancer patients, HER2‐low patients had a significantly lower pathological complete response (pCR) rate after neoadjuvant chemotherapy than HER2‐negative patients did. Zhang et al. reported that the pCR rate and recurrence rate of neoadjuvant therapy were both lower in the HER2‐low group than in the HER2‐negative group. However, another study suggested that HER2‐low breast cancer patients had a greater risk of brain metastasis than HER2‐negative patients did for HR‐positive patients [9]. In addition, a clinical drug study confirmed that trastuzumab deruxtecan (T‐DXd) treatment could reduce the risk of death by approximately 49% in HER2‐low/HR‐positive advanced patients [10]. Moreover, a phase II clinical trial over 60 months preliminarily validated the efficacy of the targeted vaccine nelipepimut‐S, which was developed for HER2‐low breast cancer, and only 5.6% of patients in the vaccine group experienced cancer recurrence compared with 25.9% in the control group [11]. These studies suggest that low HER2 expression represents a new subtype of breast cancer [12]. Therefore, effectively predicting HER2‐low breast cancer has significant clinical value.

Currently, imaging studies related to HER2‐low breast cancer are primarily based on magnetic resonance imaging (MRI) and ultrasound imaging technologies. For example, the MRI diffusion model constructed by Mao et al. demonstrated good predictive efficacy in distinguishing HER2‐low from HER2‐positive breast cancer [13]. Du et al. [14] performed a preliminary study for the diagnosis of HER2‐low breast cancer via conventional 2D grayscale ultrasound imaging combined with radiomics. Additionally, Altena et al. [15] successfully used 68Ga PET imaging to specifically visualize HER2‐low breast cancer. However, studies using multimodal ultrasound imaging to diagnose HER2‐low breast cancer are rare. Therefore, this study aimed to assist in predicting HER2‐low breast cancer via multimodal ultrasound imaging and clinicopathological indicators, providing valuable imaging information for clinical diagnosis and prognosis evaluation.

## 2 Methods

### 2.1 Patients

A total of 147 breast cancer patients at the Second Affiliated Hospital of Harbin Medical University (101 cases for training set) and Harbin Medical University Cancer Hospital (46 cases for external validation set) from September 2017 to May 2024 were finally included. All the cases were confirmed by surgical pathology. The inclusion criteria were as follows: (1) patients who underwent multimodal ultrasound examinations, including 2D ultrasound (2DUS), color Doppler flow imaging (CDFI), shear wave elastography (SWE), and contrast‐enhanced ultrasound (CEUS), before any procedures, such as biopsy or surgery; and (2) patients whose complete ultrasound imaging and clinicopathological data were available. The exclusion criteria were as follows: (1) tumors with a maximum diameter > 5 cm or minimum diameter < 5 mm (beyond the required maximum and minimum ranges for elastography examination); (2) nonmass‐type breast cancer; and (3) breast implants or breasts that underwent contrast‐enhanced ultrasound due to allergies, mental illness, or cognitive impairment. This study was approved by the Ethics Committee of the Second Affiliated Hospital of Harbin Medical University.

### 2.2 Instruments and Examination Methods

A supersonic imaging Aixplorer ultrasound diagnostic system from a French company was used for 2D US and CDFI with a high‐frequency linear array probe and for SWE and CEUS with a high‐frequency linear array probe (2–10 MHz). During the ultrasound examination, patients breathed calmly and lay supine, with both upper limbs extending outward and upward to fully expose the breasts and axillary tissue. The examination began from the nipple as the center, gradually and thoroughly scanning the entire breast. Dynamic and static scans of the tumor were conducted with 2D US and CDFI, and the images were subsequently stored. During the CDFI examination, the appropriate sample box size and blood flow velocity range were adjusted. The SWE mode was then switched on, and the sample box size and position were adjusted. After the image stabilized, it was stored. The most significant blood flow slice in the CDFI of the lesion was chosen as the observation slice for CEUS, avoiding obvious calcification and necrotic areas and retaining some normal breast tissue for comparison. The contrast agent SonoVue from Bracco was mixed with 5 mL of normal saline to form a suspension for intravenous bolus injection during the CEUS examination. The lesion was observed, and dynamic CEUS images were stored for at least 3 min. The offline data analysis software Sonoliver was used for analysis, and the time–intensity curve (TIC) and dynamic vascular pattern curve (DVPC) were automatically obtained with image fitting ≥ 75%.

### 2.3 Image Analysis and Study Parameters

Two physicians with years of experience in breast ultrasound diagnosis performed the image analysis in a double‐blind manner. In cases of discrepancies, the final result was determined by consensus. The 2D grayscale ultrasound and CDFI features, including tumor size, orientation, shape, margin, echo pattern, posterior echoes, calcification according to BI‐RADS [16], and alder blood flow grading [17], were assessed. For SWE analysis, qualitative features such as the “hard ring sign” and “black hole sign” were observed, and quantitative features, including the maximum value of the Young's modulus (Emax), mean value (Emean), minimum value (Emin), and stiffness ratio of the lesion and surrounding normal tissue (Eratio), were obtained. On CEUS, the rise time (RT, defined as the time it takes for the contrast agent to reach 10% to 90% of the peak intensity), time to peak (TTP, defined as the time from the start of contrast injection until the peak intensity is reached), mean transit time (mTT, defined as the time from the start of contrast infusion until the intensity decreases to 50% of the peak value), and maximum intensity (Imax%, defined as the ratio of the peak intensity of the tumor to the reference area peak intensity) were derived from the TIC. DVPC, was plotted with the enhancement level of the reference area on the x‐axis and the tumor enhancement level on the *y*‐axis [18].

To evaluate the reproducibility of ultrasound feature assessment, 50 patients were randomly selected from the dataset. Two physicians with rich experience in breast ultrasound diagnosis independently evaluated all the ultrasound features for inter‐observer agreement. One of the physicians re‐evaluated the same set after a 4‐week interval for intra‐observer analysis. The intraclass correlation coefficient (ICC) was used to assess the agreement of key features. The ICC values ranged from 0.842 to 0.855, indicating good to excellent consistency.

### 2.4 Pathological Immunohistochemical Analysis

According to the American Society of Clinical Oncology's (ASCO) Breast Cancer Estrogen Receptor and Progesterone Receptor Testing Guidelines and the St. Gallen Breast Cancer International Expert Consensus [19, 20], the threshold of ER and PR was 10%, with nuclear staining of > 10% considered positive and < 10% considered negative. A Ki‐67 index ≥ 20% was defined as high expression, and < 20% was defined as low expression. According to the CAP and ASCO affirm HER‐2 Testing Guidelines [21], HER‐2 negative status was defined as a score of 0 for cell membrane staining; HER‐2 low status was defined as 1+ or 2+ with no amplification by fluorescence in situ hybridization (FISH); and HER‐2 positive status was defined as a score of 3+ or 2+ with amplification detected by FISH.

### 2.5 Statistical Methods

Statistical analysis was performed using SPSS (version 26.0) and R software (version 4.2.1).

To assess whether the available sample size was sufficient to support statistically meaningful conclusions, a post hoc power analysis was conducted using the pwr.2p2n.test function in R (version 4.2.1). The effect size was estimated using Cohen's *h* based on the observed AUCs. Continuous variables were tested for normality and expressed as mean ± standard deviations (SDs) or median (IQR), with *t*‐tests or Mann–Whitney *U* tests used as appropriate. Categorical variables were compared using chi‐square tests. Variables with significant differences in univariate analysis were included in multivariate logistic regression analysis. Model performance was assessed using ROC curves. To further evaluate clinical utility and robustness, Kappa coefficients were calculated, calibration curves and decision curve analysis (DCA) were plotted, and 95% confidence intervals were estimated by bootstrapping.

## 3 Results

The result of post hoc power analysis showed that the calculated power was 89.3% in HER2‐negative/low group (*n* = 42 vs. 32, AUC = 0.876). For the HER2‐positive/low group, the power reached 99.0% (*n* = 42 vs. 27, AUC = 0.929). These results indicate that the available sample size was sufficient to support statistically meaningful conclusions.

### 3.1 General Result

In the training set, there were 32 (31.7%) HER2‐negative breast cancers, 42 (42.6%) HER2‐low tumors, and 27 (27.7%) HER2‐positive tumors. The mean age of the patients was 52.9 ± 9.8 years (range of 30–78 years). In addition, the external validation cohort comprised 46 breast cancer patients (18 were HER2‐negative, 14 were HER2‐low, and 14 were HER2‐positive cases), and the mean age of this cohort was 55.3 **±** 7.2 years.

### 3.2 Univariate Analysis of Clinical and Pathological Indicators

In the HER2‐negative/low breast cancer group, there were statistically significant differences in the expression of Ki‐67 and PR (*p* < 0.05). In the HER2‐positive/low group, there were statistically significant differences in the expression of Ki‐67, ER, and PR (*p* < 0.05). Patient age, histological grade, and lymph node metastasis were not significantly different (*p* > 0.05), as shown in Table 1.

**TABLE 1:** Univariate analysis of clinicopathological and two‐dimensional ultrasound features.

Group	HER2‐negative (*n* = 32)	HER2‐low (*n* = 42)	*t*‐valu*e*/z‐value/*χ* ^2^	*p*	HER2‐positive (*n* = 27)	HER2‐low (*n* = 42)	*t*‐value/*z*‐value/*χ* ^2^	*p*
Age	51 ± 10 years	54 ± 8 years	1.034	0.14	52 ± 10 years	54 ± 8 years	1.500	0.302
Histological grade			1.788	0.372			1.499	0.316
(I/II)	20 (62.5%)	32 (76.2%)			19 (70.4%)	32 (76.2%)		
(III)	12 (37.5%)	10 (23.8%)			8 (29.6%)	10 (23.8%)		
Lymph node metastasis			0.001	0.574	0.014	0.907	0.014	0.907
Yes	8 (25.0%)	29 (69.0%)			8 (29.6%)	29 (69.0%)		
No	24 (75.0%)	13 (31.0%)			19 (70.4%)	13 (31.0%)		
Ki‐67			13.663	0.000			11.605	0.001
High	23 (71.9%)	12 (28.6%)			19 (70.4%)	12 (28.6%)		
Low	9 (28.1%)	30 (71.4%)			8 (29.6%)	30 (71.4%)		
ER			4.131	0.051			4.940	0.030
Positive	20 (62.5%)	35 (83.3%)			16 (59.3%)	35 (83.3%)		
Negative	12 (37.5%)	7 (16.7%)			11 (40.7%)	7 (16.7%)		
PR			9.837	0.002			11.051	0.001
Positive	12 (37.5%)	31 (73.8%)			18 (66.7%)	31 (73.8%)		
Negative	20 (62.5%)	11 (26.2%)			9 (33.3%)	11 (26.2%)		
Tumor size			0.643	0.423			2.799	0.098
< 2 cm	18 (56.3%)	21 (50.0%)			19 (70.4%)	21 (50.0%)		
≥ 2 cm	14 (43.8%)	21 (50.0%)			8 (29.6%)	21 (50.0%)		
Orientation			1.196	0.279			0.097	0.755
Parallel	27 (84.4%)	31 (73.8%)			19 (70.4%)	31 (73.8%)		
Vertical	5 (15.6%)	11 (26.2%)			8 (29.6%)	11 (26.2%)		
Shape			5.55	0.999			1.578	1.000
Regular	4 (12.5%)	0 (0.00%)			1 (3.7%)	0 (0.00%)		
Irregular	28 (87.5%)	42 (100%)			26 (96.3%)	42 (100%)		
Margin			1.309	0.254			0.749	0.388
Non‐circumscribed	21 (65.6%)	22 (52.4%)			17 (63.0%)	22 (52.4%)		
Circumscribed	11 (34.6%)	20 (47.6%)			10 (37.0%)	20 (47.6%)		
Echo pattern			2.503	0.117			4.684	0.033
Hypoechoic	14 (43.8%)	31 (73.8%)			13 (48.1%)	31 (73.8%)		
Mixed echo	18 (56.2%)	11 (26.2%)			14 (51.9%)	11 (26.2%)		
Posterior echo			2.988	0.864			0.785	0.413
No change	15 (46.9%)	21 (50.0%)			16 (59.3%)	21 (50.0%)		
Attenuation	13 (40.6%)	20 (47.6%)			10 (37.0%)	20 (47.6%)		
Enhancement	4 (12.5%)	1 (2.4%)			1 (3.7%)	1 (2.4%)		
Calcification			1.049	0.307			0.519	0.472
Yes	16 (50.0%)	26 (61.9%)			19 (70.4%)	26 (61.9%)		
No	16 (50.0%)	16 (38.1%)			8 (29.6%)	16 (38.1%)		
Alder			0.903	0.535			64.592	0.198
0–I grade	9 (28.1%)	10 (23.8%)			3 (11.1%)	10 (23.8%)		
II–III grade	23 (71.9%)	32 (76.2%)			24 (83.9%)	32 (76.2%)		

*Note:* The numbers in parentheses represent the percentage. Ki‐67 refers to nuclear proliferation‐related protein; ER refers to estrogen receptor; PR refers to progesterone receptor.

### 3.3 Univariate Analysis of Multimodal Ultrasound Features

In the HER2‐negative/low breast cancer group, no statistically significant differences were found in any of the 2D grayscale or CDFI features (*p* > 0.05). However, in the HER2‐positive/low group, there were statistically significant differences in internal echo features (*p* < 0.05), as shown in Table 1.

In the HER2‐negative/low group, the Emax and Emean values were significantly different (*p* < 0.05) on SWE. In the HER2‐positive/low group, the Emax, Emean, Emin, and Eratio values were significantly different (*p* < 0.05). There were no statistically significant differences in “hard ring” or “black hole” features (*p* > 0.05), as shown in Table 2.

**TABLE 2:** Quantitative and qualitative feature analysis of SWE features.

Group	HER2‐negative (*n* = 32)	HER2‐low (*n* = 42)	*t*‐value/*z*‐value/χ^2^	*p*	HER2‐positive (*n* = 27)	HER2‐low (*n* = 42)	*t*‐value/*z*‐value/*χ* ^2^	*p*
Emax	130.1 (94.1, 150.8)	80.8 (64.0, 122.4)	−3.224	0.006	162.8 (125.8, 186.1)	80.8 (64.0, 122.4)	−4.998	0.000
Emean	86.8 (64.2, 105.4)	60.0 (50.7, 98.4)	−2.122	0.035	125.1 (81.4, 146.1)	60.0 (50.7, 98.4)	−4.254	0.000
Emin	51.0 (21.0, 74.0)	41.3 (18.1, 63.1)	−0.884	0.345	56.2 (28.7, 99.4)	41.3 (18.1, 63.1)	−2.004	0.022
Eratio	11.0 (7.1, 15.0)	9.6 (6.2, 17.6)	−0.005	0.553	15.6 (9.6, 29.2)	9.6 (6.2, 17.6)	−2.183	0.014
Black hole sign			2.171	0.164			0.288	0.592
Present	20 (62.5)	19 (45.2)			14 (51.9)	19 (45.2)		
Absent	12 (37.5)	23 (54.8)			13 (48.1)	23 (54.8)		
Hard ring sign			0.759	0.438			2.069	0.153
Present	25 (78.1)	29 (69.0)			14 (51.9)	29 (69.0)		
Absent	7 (21.9)	13 (31.0)			13 (48.1)	13 (31.0)		

*Note:* The numbers in parentheses represent percentages and the first and third quartiles. Emax: Maximum Young's Modulus; Emean: Average Value; Emin: Minimum Value; Eratio: Ratio of the stiffness between the lesion and surrounding normal tissue.

In terms of CEUS features, there were statistically significant differences in tumor enhancement level, rise time (RT), and time to peak (TTP) (*p* < 0.05) in the HER2‐negative/low group, and RT and TTP between the HER2‐positive/low group (*p* < 0.05). No statistically significant differences were found in the qualitative features, such as filling defects, homogeneity, branching vessels, or post enhancement. However, (DVPC) was significantly different among the different groups (*p* < 0.05), as shown in Table 3.

**TABLE 3:** Quantitative and qualitative analysis of CEUS features.

Group	HER2‐negative (*n* = 32)	HER2‐low (*n* = 42)	*t*‐value/*z*‐value/*χ* ^2^	*p*	HER2‐positive (*n* = 27)	HER2‐low (*n* = 42)	*t*‐value/*z*‐value/*χ* ^2^	*p*
IMAX	77.3 (37.2, 129.3)	56.1 (34.4, 104.8)	−1.157	0.672	83.5 (36.7, 126.8)	56.1 (34.4, 104.8)	−1.021	0.654
RT	7.9 (7.2, 9.7)	9.3 (7.0, 14.7)	−1.91	0.016	6.6 (5.3, 9.8)	9.3 (7.0, 14.7)	−2.496	0.038
TTP	11.2 (9.4, 13.1)	11.0 (9.0, 19.1)	−0.633	0.047	8.7 (6.9, 12.0)	11.0 (9.0, 19.1)	−2.472	0.034
MTT	36.4 (20.1, 74.6)	32.8 (17.9, 59.9)	−1.157	0.262	18.5 (14.0, 31.6)	32.8 (17.9, 59.9)	−1.973	0.235
Filling defects			0.548	0.460			3.734	0.056
Present	11 (34.4%)	18 (42.9%)			18 (66.7%)	18 (42.9%)		
Absent	21 (65.6%)	24 (57.1%)			9 (33.3%)	24 (57.1%)		
Uniformity			1.170	0.281		3.648		0.062
Uniform	15 (46.9%)	25 (59.5%)			22 (81.5%)	25 (59.5%)		
Non‐uniform	17 (53.1%)	17 (40.5%)			5 (18.5%)	17 (40.5%)		
Perforating vessels			1.945	0.166			1.503	0.544
Present	8 (25.0%)	17 (40.5%)			15 (55.6%)	17 (40.5%)		
Absent	24 (75.0%)	25 (59.5%)			12 (44.4%)	25 (59.5%)		
Enhanced area			0.001	0.974			3.411	0.097
Enlarged	6 (18.8%)	8 (19.0%)			1 (3.7%)	8 (19.0%)		
Not enlarged	26 (81.3%)	34 (81.0%)			26 (96.3%)	34 (81.0%)		
Enhancement level			13.278	0.000			4.657	0.065
Low	17 (53.1%)	38 (90.5%)			20 (71.1%)	38 (90.5%)		
Equal/High	15 (46.9%)	4 (9.5%)			7 (19.9%)	4 (9.5%)		
DVPC wave direction			15.100	0.001			13.964	0.001
Forward	11 (34.3%)	10 (23.8%)			15 (55.6%)	10 (23.8%)		
Reverse	17 (53.1%)	9 (21.4%)			9 (33.3%)	9 (21.4%)		
Forward‐then‐reverse	4 (12.5%)	20 (47.6%)			3 (11.1%)	20 (47.6%)		
Reverse‐then‐forward	0 (0.0%)	3 (7.1%)			0 (0.0%)	3 (7.1%)		

*Note:* The numbers in parentheses represent the percentage and the first and third quartiles. RT refers to the time for contrast agent infusion from 10% to 90% peak intensity; TTP refers to the time from contrast injection to peak intensity; MTT refers to the time from contrast infusion to decay to 50% of peak intensity; IMAX refers to peak intensity; DVPC refers to dynamic vascular model curve.

### 3.4 Multivariate Analysis of Clinical Pathological and Multimodal Ultrasound Features

Multivariate logistic regression analysis revealed that Ki‐67, PR, Emax, and RT were independent factors in the HER2‐negative/low group. Patients with HER2‐low breast cancer tended to have low Ki‐67 expression, positive PRs, low Emax values, and long RTs (Table 5).

**FIGURE 1:**
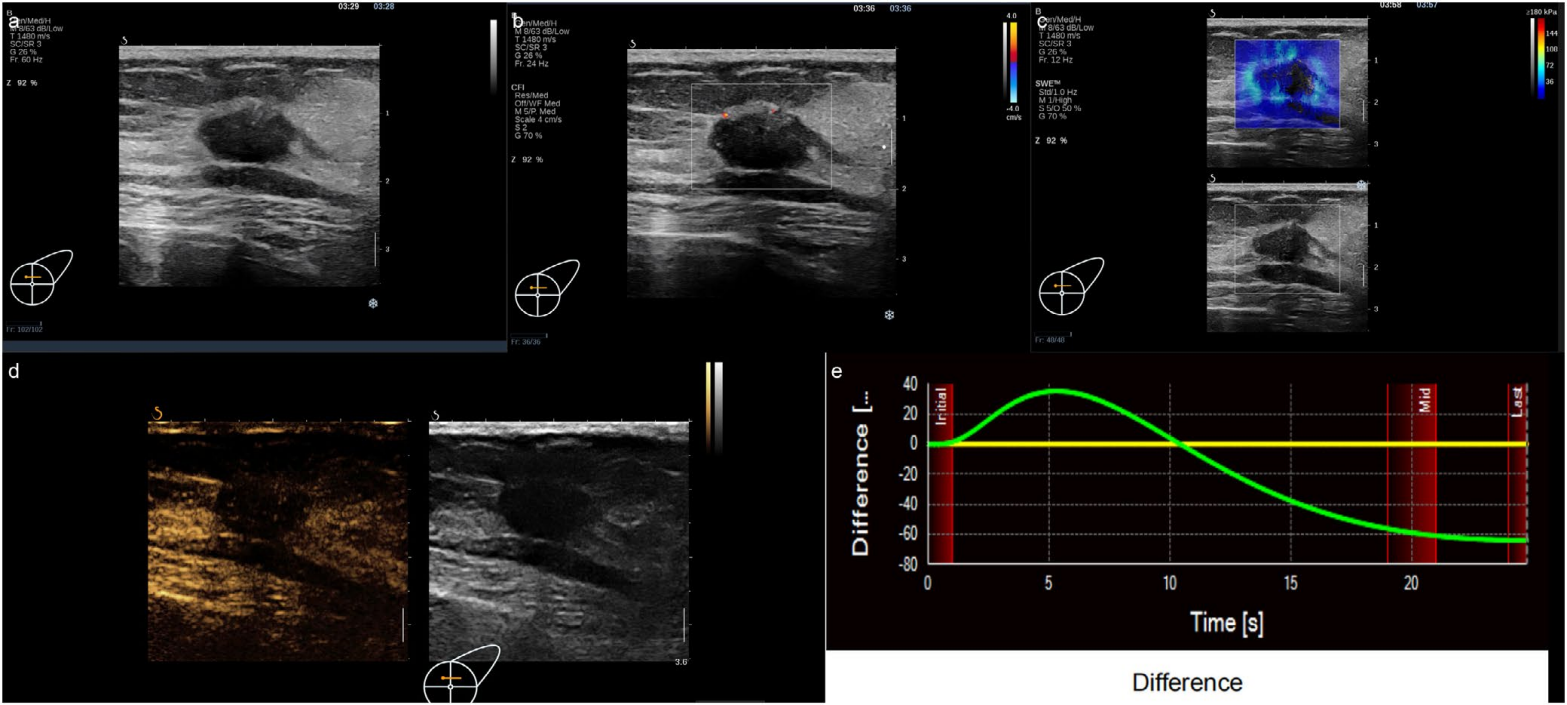
Multi‐modal ultrasound images of HER‐2 low breast cancer. The mass shows irregular shape, lobulated margins, calcifications, and Grade I flow. SWE reveals a black hole sign. CEUS demonstrates heterogeneous centripetal enhancement with a biphasic DVPC wave (initial rise followed by rapid washout).

**FIGURE 2:**
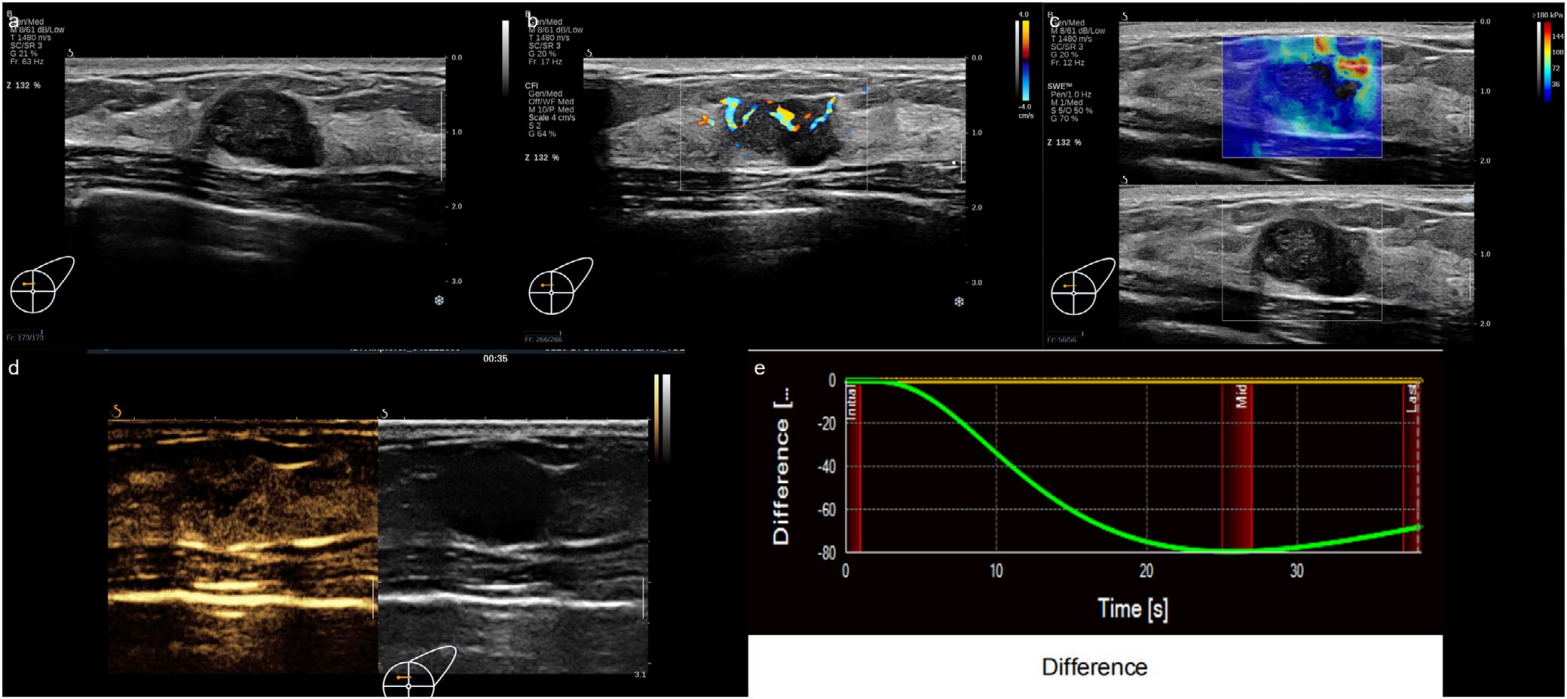
Multi‐modal ultrasound images of HER‐2 negative breast cancer. The lesion presents with irregular shape, enhanced posterior echoes, and Grade III flow. SWE shows a stiff ring sign. CEUS shows low overall enhancement and a negative DVPC wave pattern.

**FIGURE 3:**
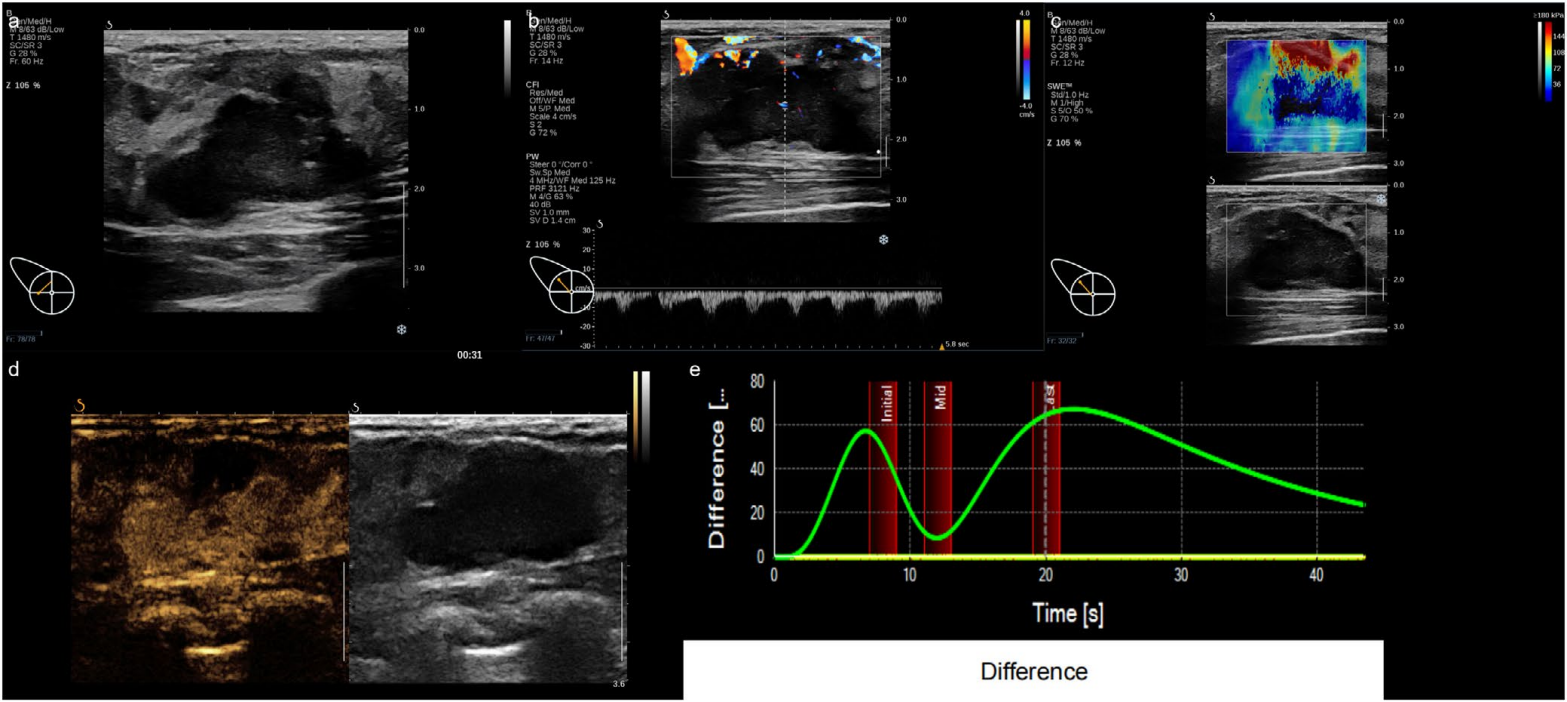
Multimodal ultrasonographic findings in HER‐2 positive breast cancer. The hypoechoic mass displays lobulated margins, calcifications, and Grade III flow. SWE reveals a stiff ring sign. CEUS shows persistent high enhancement with filling defects and a consistently elevated DVPC curve.

Multivariate analysis revealed Ki‐67, ER, and PR expression, the Emax value, and the DVPC as independent predictors in the HER2‐positive/low group. Patients with low Ki‐67, positive ER and PR, low Emax values, and a DVPC curve of initially positive and then negative wave patterns were more likely to exhibit low HER2 expression (Table 6 and Figures 1, 2, 3).

**FIGURE 4:**
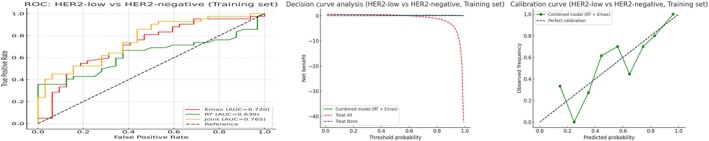
Model performance for distinguishing HER2‐low from HER2‐negative in the training cohort (combination of Emax and RT). From left to right: ROC curve, decision curve analysis (DCA), and calibration curve. The model demonstrates high discriminative ability and clinical net benefit, with good calibration in the training dataset.

**FIGURE 5:**
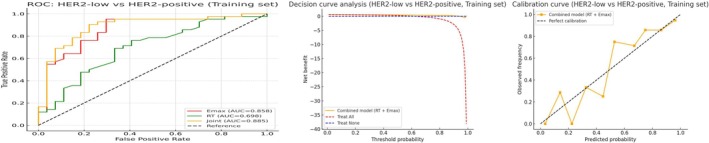
Model performance for distinguishing HER2‐low from HER2‐positive in the training cohort (combination of Emax and RT). From left to right: ROC curve, DCA, and calibration curve. The model shows strong performance in internal validation, with well‐calibrated prediction probabilities.

**FIGURE 6:**
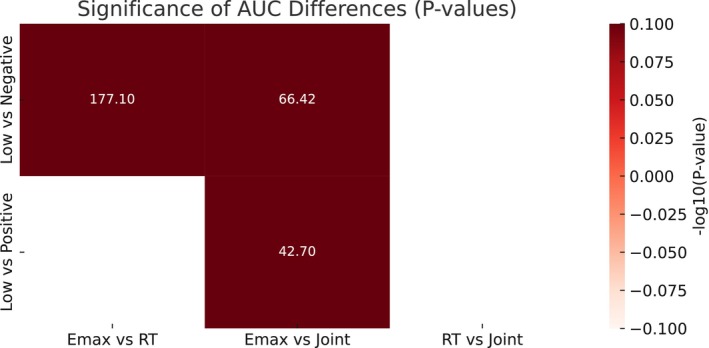
Statistical significance of AUC differences across feature combinations. Heatmap visualizes DeLong's test results for AUC comparisons among models using Emax, RT, or both. Higher −log10(*p*) values indicate more significant differences, stratified by HER2 expression subtype.

**FIGURE 7:**
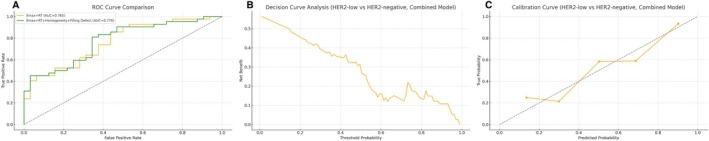
The model (Figures 11 and 12) was constructed as an exploratory analysis combining qualitative and quantitative ultrasound features to differentiating HER2‐low from HER2‐negative in train set. (A) ROC analysis shows a slight improvement in AUC with the combined model (0.776 vs. 0.765). (B) DCA confirms comparable net clinical benefit. (C) Calibration curve of the combined model indicates improved probability estimation accuracy.

**FIGURE 8:**
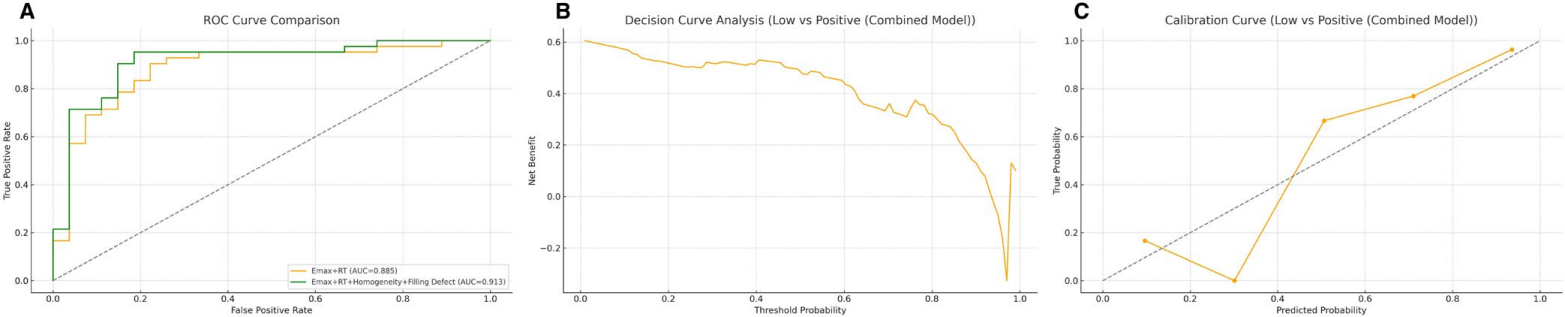
Comparison of the quantitative model and the combined model for differentiating HER2‐low from HER2‐positive in the train set. (A) ROC curve shows an increase in AUC from 0.885 (quantitative model) to 0.913 (combined model). (B) DCA supports enhanced clinical utility with the combined approach. (C) The calibration curve of the combined model reveals improved consistency between predicted and actual outcomes.

Given that the interaction between different parameters for capturing distinct aspects of tumor biology and providing complementary information, we also explore whether combining the quantitative feature of Emax and RT (both were statistically significant in multivariate analysis) could improve the diagnostic performance compared to a single feature (Table 4 and Figures 4, 5, 6). The combination of Emax and RT consistently outperformed the individual feature with the higher AUCs (0.765 vs. 0.720/0.630, *p* = 0.009/< 0.001), sensitivity, and specificity. The *F*1 scores and Kappa values of combined Emax and RT were 0.780 and 0.429 in the HER2 negative/low group, and 0.880 and 0.686 in the positive/low group. However, for qualitative features (combination of perfusion defect and homogeneity), there was limited discriminative ability in diagnosis with the AUC and specificity, respectively, of 0.589/0.313 in the HER2‐low/negative group and 0.690/0.556 in the HER2‐low/positive group. When they further combined with quantitative parameters (Emax and RT), the AUC and specificity improved to 0.776/0.633 in the HER2‐low/negative group and 0.913/0.822 in the HER2‐positive/low group (Table 8 and Figures 7 and 8).

**TABLE 4:** Performance comparison based on Emax and RT features.

Group	Feature	AUC	*p*	Sensitivity	Specificity	PPV	NPV	*F*1‐score	Kappa
HER2‐negative/low	Emax	0.720 (0.594 to 0.827)	0.0091	0.760 (0.632 to 0.887)	0.531 (0.351 to 0.699)	0.681 (0.557 to 0.807)	0.630 (0.432 to 0.792)	0.719 (0.614 to 0.837)	0.299 (0.080 to 0.503)
	RT	0.630 (0.514 to 0.759)	< 0.001	0.667 (0.533 to 0.807)	0.531 (0.369 to 0.722)	0.651 (0.483 to 0.785)	0.548 (0.362 to 0.714)	0.659 (0.533 to 0.765)	0.199 (−0.033 to 0.339)
	Combined Emax and RT	0.765 (0.653 to 0.867)		0.860 (0.750 to 0.952)	0.560 (0.387 to 0.731)	0.655 (0.523 to 0.780)	0.452 (0.443 to 0.720)	0.780 (0.682 to 0.865)	0.429 (0.223 to 0.630)
HER2‐positive/low	Emax	0.858 (0.740 to 0.942)	0.0059	0.905 (0.802 to 0.977)	0.704 (0.531 to 0.863)	0.826 (0.702 to 0.920)	0.826 (0.627 to 0.962)	0.864 (0.789 to 0.930)	0.625 (0.415 to 0.808)
	RT	0.698 (0.568 to 0.840)	0.0246	0.833 (0.721 to 0.937)	0.370 (0.197 to 0.563)	0.673 (0.547 to 0.792)	0.588 (0.349 to 0.802)	0.745 (0.655 to 0.847)	0.218 (−0.001 to 0.424)
	Combined Emax and RT	0.885 (0.790 to 0.961)		0.901 (0.795 to 0.977)	0.780 (0.619 to 0.920)	0.786 (0.662 to 0.938)	0.333 (0.363 to 0.695)	0.880 (0.800 to 0.946)	0.686 (0.501 to 0.851)

*Note:* The combination of Emax and RT significantly improved diagnostic performance compared to either parameter (all *p* < 0.001). In this table, all confidence intervals were estimated using bootstrapping with 1000 resamples.

Abbreviations: AUC, area under the ROC curve; NPV, negative predictive value; PPV, positive predictive value.

**TABLE 5:** Multivariate analysis for HER2‐negative/low breast cancer.

Feature	*B* value	SE value	Wald *χ* ^2^	*p*	OR (95% CI)
ki‐67	−1.762	0.703	6.282	0.012	0.172 (0.043–0.681)
PR	1.866	0.741	6.34	0.012	6.465 (1.515–27.639)
Emax	−0.03	0.015	4.31	0.038	0.97 (0.943–0.998)
Emean	0.024	0.02	1.418	0.234	1.024 (0.985–1.066)
TTP	−0.294	0.208	1.992	0.158	0.745 (0.496–1.121)
RT	0.559	0.282	3.922	0.048	1.749 (1.006–3.042)
Enhancement level	−1.031	0.796	1.678	0.195	0.357 (0.075–1.697)
DVPC type	0.682	0.461	2.186	0.139	1.978 (0.801–4.888)
Constant	−0.948	1.944	0.238	0.626	0.388

*Note:* The numbers in parentheses represent the 95% confidence intervals. Only variables identified as significant in univariate analysis (*p* < 0.05) were included in the multivariate analysis.

**TABLE 6:** Multivariate analysis of HER2‐positive/low breast cancer.

Feature	*B* value	SE value	Wald *χ* ^2^	*p*	OR (95% CI)
ki‐67	−4.926	2.111	5.443	0.020	0.007 (0–0.455)
PR	7.933	3.135	6.401	0.011	2787.189 (5.973–1,300,487)
ER	−5.338	2.527	4.463	0.035	0.005 (0–0.68)
Internal echo	−1.436	1.761	0.665	0.415	0.238 (0.008–7.498)
DVPC type	3.103	1.554	3.989	0.046	22.268 (1.06–468.007)
Eratio	−0.075	0.075	1.003	0.317	0.928 (0.801–1.075)
Emin	0.044	0.045	0.936	0.333	1.045 (0.956–1.142)
Emean	0.01	0.05	0.045	0.833	1.011 (0.917–1.114)
Emax	−0.094	0.042	5.031	0.025	0.911 (0.839–0.988)
TTP	0.466	0.715	0.426	0.514	1.594 (0.35–4.474)
RT	0.339	0.757	0.201	0.654	0.654 (0.319–6.187)
Constant	2.729	3.447	0.626	0.429	15.311

*Note:* The numbers in parentheses represent the 95% confidence intervals. Only variables identified as significant in univariate analysis (*p* < 0.05) were included in the multivariate analysis.

**TABLE 7:** Diagnostic performance of the models in the training set and external validation set.

Group	AUC	Sensitivity	Specificity	PPV	NPV	*F*1‐score	Kappa
HER2‐negative/low (training)	0.876 (0.780–0.953)	0.833 (0.718–0.933)	0.781 (0.631–0.920)	0.833 (0.717–0.937)	0.781 (0.066–0.282)	0.833 (0.742–0.911)	0.614 (0.427–0.791)
Validation set	0.925 (0.806–0.998)	0.857 (0.643–1.000)	0.888 (0.714–0.990)	0.857 (0.633–0.932)	0.888 (0722–0.976)	0.857 (0.690–0.971)	0.746 (0.475–0.938)
HER2‐positive/low(training)	0.929 (0.855–0.978)	0.905 (0.805–0.977)	0.856 (0.559–0.880)	0.844 (0.725–0.932)	0.833 (0.023–0.195)	0.868 (0.785–0.933)	0.647 (0.442–0.800)
Validation set	0.918 (0.789–1.000)	0.714 (0.444–0.929)	0.785 (0.538–1.000)	0.769 (0.500–0.893)	0.733 (0.500–0.938)	0.740 (0.500–0.889)	0.610 (0.156–0.784)

*Note:* AUC refers to the area under the ROC curve. Sensitivity, specificity, positive predictive value (PPV), negative predictive value (NPV), and *F*1‐score represent the diagnostic performance of the model. Kappa measures inter‐rater agreement beyond chanc. Inthis table, all confidence intervals were estimated using bootstrapping with 1000 resamples.

**TABLE 8:** Diagnostic performance of logistic regression models based on qualitative and quantitative features for HER2‐low breast cancer.

Group	Feature	AUC	*p*	Sensitivity	Specificity	ppv	npv	*F*1‐score	Kappa
HER2‐negative/low group	Qualitative	0.589 (0.464 to 0.710)		0.810 (0.692 to 0.925)	0.313 (0.156 to 0.478)	0.607 (0.463 to 0.727)	0.556 (0.333 to 0.800)	0.694 (0.575 to 0.785)	0.129 (−0.072 to 0.329)
	Combined	0.776 (0.689 to 0.889)	0.005	0.767 (0.548 to 0.920)	0.633 (0.357 to 0.854)	0.713 (0.571 to 0.833)	0.655 (0.512 to 0.786)	0.748 (0.590 to 0.863)	0.407 (0.162 to 0.629)
HER2‐positive/low group	Qualitative	0.690 (0.567 to 0.803)		0.786 (0.652 to 0.897)	0.556 (0.367 to 0.737)	0.733 (0.595 to 0.854)	0.625 (0.435 to 0.813)	0.759 (0.649 to 0.847)	0.348 (0.117 to 0.563)
	Combined	0.913 (0.843 to 0.980)	< 0.001	0.911 (0.795 to 0.979)	0.822 (0.611 to 0.941)	0.890 (0.792 to 0.961)	0.863 (0.731 to 0.962)	0.900 (0.805 to 0.963)	0.740 (0.529 to 0.905)

*Note:* The Emax + RT model results are not displayed separately in this table. Please refer to Table 4 for details, as they are already included in the combined model performance. *p*‐values indicate the statistical significance of the AUC improvement between the qualitative and combined models, based on bootstrap comparison. *p*‐values for AUC improvement were calculated in the training cohort. In this table, all confidence intervals were estimated using bootstrapping with 1000 resamples.

### 3.5 Model Evaluation and Validation

**FIGURE 9:**
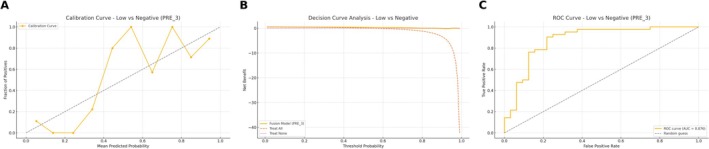
Training performance of the fusion model for differentiating HER2‐low from HER2‐negative. (A) Calibration curve demonstrates reasonable prediction reliability. (B) DCA shows clinical net benefit over default strategies. (C) ROC curve yields an AUC of 0.876, indicating solid classification performance in the training dataset.

**FIGURE 10:**
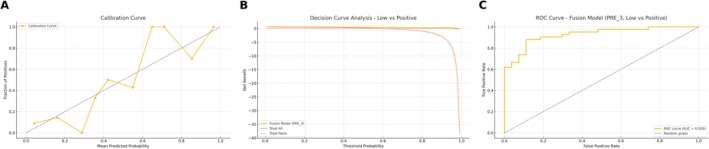
Training performance of the fusion model for differentiating HER2‐low from HER2‐positive. (A) Calibration curve indicates strong agreement between predicted probabilities and observed outcomes in the training set. (B) Decision curve analysis (DCA) shows substantial net benefit across decision thresholds. (C) The ROC curve exhibits an AUC of 0.929, reflecting excellent discrimination and model fitting within the internal cohort.

**FIGURE 11:**
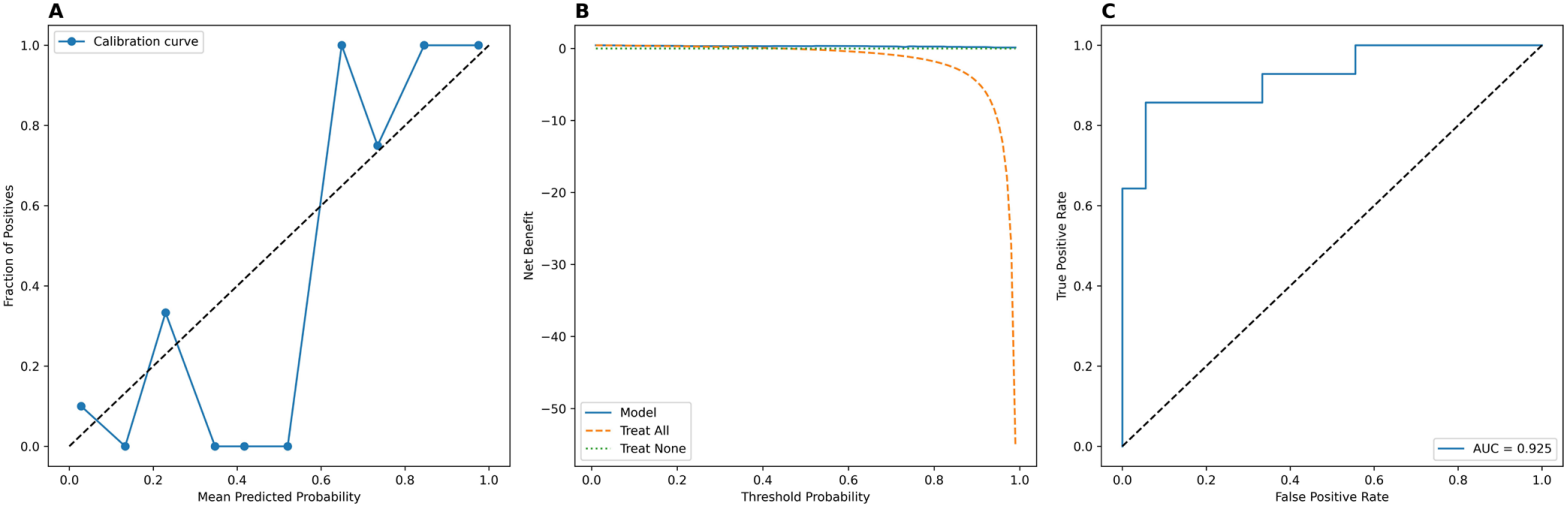
External validation of the fusion model for differentiating HER2‐low from HER2‐positive. (A) Calibration curve shows strong agreement between predicted and observed probabilities. (B) Decision curve analysis (DCA) indicates consistent net clinical benefit over a wide range of thresholds. (C) ROC curve demonstrates excellent discriminative power with an AUC of 0.925 in the external cohort.

**FIGURE 12:**
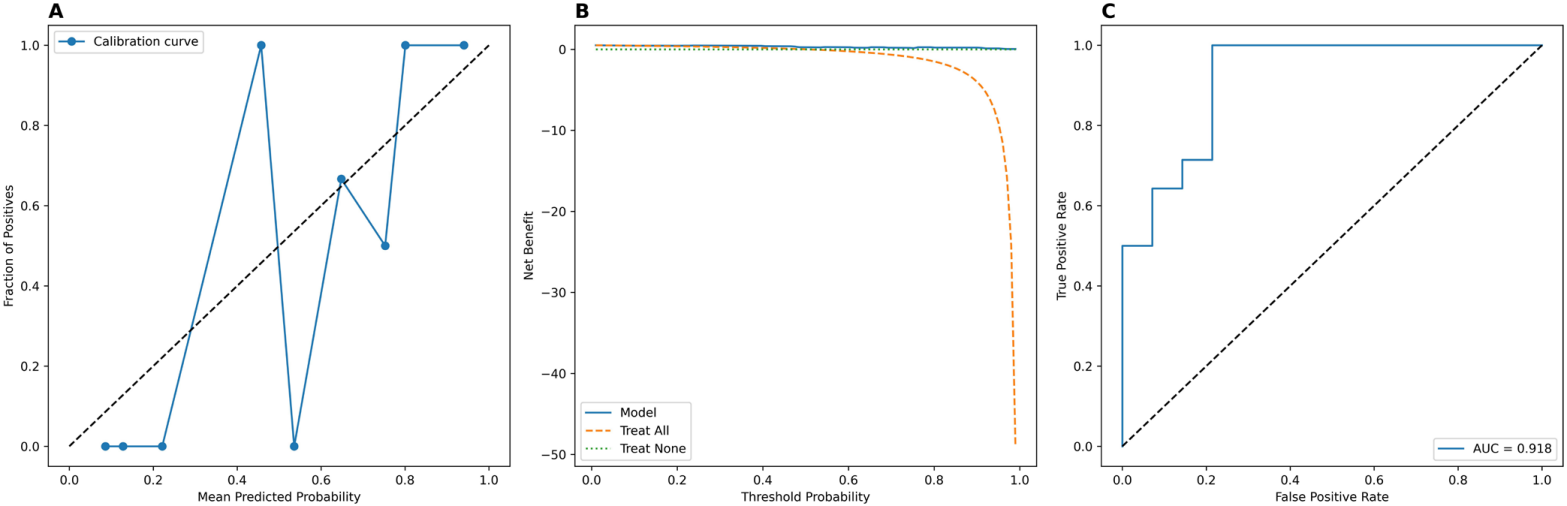
External validation of the fusion model for differentiating HER2‐low from HER2‐negative. (A) Calibration curve demonstrates good calibration of predicted probabilities. (B) DCA reveals favorable clinical utility across varying decision thresholds. (C) The ROC curve shows an AUC of 0.918, confirming the model's generalizability and robust diagnostic performance in external data.

A logistic regression model was developed based on multimodal ultrasound and clinicopathological feature. For the training set of the HER2‐negative/low group, the AUC of the diagnostic model was 0.876 (95% CI = 0.780–0.953), the sensitivity was 0.833, the specificity was 0.781 (Kappa = 0.614, *p* < 0.001), with an *F*1 score of 83.3% (Table 7). For the HER2‐positive/low group, the AUC was 0.929 (95% CI = 0.855–0.978), the sensitivity was 0.905, the specificity was 0.856 (Kappa = 0.647, *p* < 0.001), with an *F*1 score of 86.8% (Table 7 and Figures 9 and 10). In the external validation set, the HER2‐negative/low model yielded excellent performance with the AUC of 0.925 (95% CI: 0.806–0.998), and the HER2‐positive/low model achieved an AUC of 0.918 (95% CI: 0.789–1.000). Detailed data are shown in Table 7. The DCA and calibration curves demonstrated favorable clinical utility and prediction consistency (Figures 11 and 12).

## 4 Discussion

The HER2 gene is an oncogene located on the long arm of chromosome 17 (17q21) and encodes a transmembrane protein belonging to the epidermal growth factor receptor family. The HER2 protein is expressed at low levels in normal breast tissue and plays an important role in the processes of cell growth, differentiation, and survival. Approximately15 to 20% of breast cancer tumors exhibit HER2 gene (protein) amplification or overexpression, which is associated with high tumor invasiveness and poor prognosis [22]. Another large sample study confirmed that HER2‐low cases account for approximately 45% to 55% of all breast cancers [23]. There are variations in data across regions, and HER2‐low cases account for approximately 30% to 44% of cases in China [4]. Previously, patients with low HER2 expression were often considered the negative group, and the treatment method was based on HR status. At present, the novel antibody–drug conjugate T‐DXd is a targeted drug for treating HER2‐low breast cancer. In a clinical drug trial cohort study of DESTINY‐Breast04, T‐DXd significantly improved the progression‐free survival (PFS) and overall survival (OS) of HER2‐low metastatic patients [10]. Furthermore, molecular biology studies revealed that, compared with HER2‐positive breast cancer, HER2‐low tumors presented significant increases in mutations in genes such as PTEN, GATA3, CBFB, and Akt1, and compared with HER2‐negative cases, HER2‐low tumors presented significantly greater mutation rates in the CBFB, PIK3CA, MAP3K1, and ARID1A genes, particularly in genes related to the PI3K‐Akt signaling pathway [6]. These findings suggest that HER2‐low breast cancer is a distinct subtype.

As a distinct molecular subtype, accurate identification of HER2‐low breast cancer holds important therapeutic and prognostic implications. Unlike HER2‐negative patients, who are not candidates for HER2‐targeted therapy, and HER2‐positive patients, who benefit from established anti‐HER2 treatments such as trastuzumab and pertuzumab, those with HER2‐low expression may now derive clinical benefit from novel antibody‐drug conjugates (ADCs). Notably, the DESTINY‐Breast04 trial demonstrated significantly improved progression‐free and overall survival in HER2‐low patients treated with trastuzumab deruxtecan (T‐DXd). In contrast, patients with HER2‐0 expression (IHC 0), representing a true HER2‐negative population, currently have no approved HER2‐targeted therapeutic options and remain reliant on chemotherapy or endocrine therapy if hormone receptor–positive. This highlights an urgent unmet need for developing novel targeted strategies for this subgroup. Therefore, precise determination of HER2‐low status not only enhances molecular classification but also expands treatment opportunities and may improve outcomes for a subgroup previously considered untreatable with HER2‐targeted agents.

While core needle biopsy and immunohistochemistry (IHC) remain the gold standard for determining HER2 status, these methods have limitations, including sampling error, intratumoral heterogeneity, tissue quality constraints, and interpretive variability. In clinical settings where biopsy is infeasible or yields inconclusive results, a non‐invasive imaging‐based approach could serve as a complementary tool to pre‐screen patients, assess HER2 expression heterogeneity, and support treatment decision‐making. In this context, our study explores the potential role of contrast‐enhanced ultrasound (CEUS)‐based parameters to aid in the early identification of HER2‐low tumors prior to or alongside pathological confirmation, thereby providing valuable support for individualized treatment planning. In this study, multimodal ultrasound imaging combined with clinicopathological information could be effectively used to predict HER2‐low breast cancer, providing valuable imaging‐based evidence for clinical diagnosis, treatment, and prognosis assessment.

### 4.1 Clinical Pathological Features and HER2‐Low Breast Cancer

Compared with HER2‐low breast cancer, HER2‐negative tumors are associated with higher mutation rates in tumor suppressor genes and proto‐oncogenes such as TP53, TERT, GALNT12, CARD11, and TRRAP, which promote extensive tumor cell proliferation [6]. Additionally, when HER2 is positively expressed [24], the RAS‐RAF‐MEK‐ERK pathway, which mediates cell proliferation, is activated, leading to rapid tumor cell proliferation. As a result, HER2‐low breast cancer patients tend to have lower Ki‐67 indices. In this study, HER2‐low breast cancer patients were more likely to be HR positive than HER2‐positive patients were, which was consistent with the findings of Schettini [25]. Several cohort studies have suggested that the estrogen receptor 1 (ESR1) gene, encoding estrogen receptor alpha (ERα), has a relatively high mutation rate in HER2‐low tumors [26]. The constitutive transcriptional activity of the mutated ESR1 gene ligand could activate ERα even without estrogen binding, leading to a high percentage of ER‐specific cells (more than 90% of cells are typically strongly positive). Furthermore, ER positivity can promote cell proliferation and carcinogenesis by activating signaling pathways such as the phosphoinositide 3‐kinase alpha (PIK3CA) and mitogen‐activated protein kinase (MAPK) pathways. PR is a target gene of the ER, and PR expression depends on estrogen levels. As a transcription factor, the ER recruits the transcription complex by binding to estrogen response elements (EREs) on the PR gene, thus activating the transcription of the PR gene. Additionally, the ER can further promote PR expression via pathways such as the RAS‐RAF‐MEK‐ERK and PI3K‐AKT‐mTOR pathways. Furthermore, HER2‐low patients were more likely to be PR positive than HER2‐negative patients were, which was consistent with the results of Zhang [6] and others.

### 4.2 Relationships Between SWE and CEUS Features and HER2‐Low Breast Cancer

SWE provides stiffness information for breast tumors. In previous studies, such as those by Yan [27], SWE could improve the ultrasound diagnosis of breast masses from benign to malignant, increasing the diagnostic efficacy from 0.772 to 0.957 via both quantitative and qualitative parameters. This study revealed that the Emax values of HER2‐low breast cancer patients were significantly lower than those of HER2‐positive and HER2‐negative patients (*p* < 0.001). HER2‐positive breast cancers are pathologically characterized by rapid tumor cell proliferation, increased tissue density, adhesive forces, and matrix stiffness. Additionally, HER2‐positive tumors can activate fibroblast and matrix metalloproteinase (MMP) expression through various signaling pathways, such as the PI3K/AKT and MAPK pathways, promoting extracellular matrix remodeling and collagen fiber deposition [28], thus resulting in increased stiffness. Conversely, HER2‐negative tumors often exhibit low Ki‐67 expression due to poor blood supply and hypoxic conditions that further activate tumor signaling pathways (such as the HIF‐1α pathway), leading to the activation of fibrogenic factors such as TGF‐β, promoting the transformation of fibroblasts and increasing tissue stiffness through the synthesis of large amounts of collagen [29]. In addition, hypoxia inhibits the degrading action of matrix metalloproteinases, further increasing tissue stiffness.

HER‐2 genes stimulate the formation of new blood vessels in tumors [24]. In this study, SonoLiver software was used for offline quantitative analysis of contrast images, performing motion compensation during the imaging process to minimize interference from breathing movements and more accurately reflecting the tumor's hemodynamic characteristics numerically [30]. In this study, the RT of HER2‐low patients was longer than that of HER2‐negative patients (*p* < 0.05). However, Wang and others reported no statistically significant difference in the rising slope (K), which represents the duration of RT, between these two groups [31]. Since 71.9% of the HER2‐negative breast cancers in our study presented high Ki‐67 expression and HR negativity, these tumors were highly invasive and compressed surrounding tissues during infiltration, which altered hemodynamics and remodeled vessels, accelerating the transit time of the contrast agent. Additionally, rapid tumor cell proliferation can lead to the concurrence of hypoxia [32], thereby activating hypoxia‐inducible factor (HIF) signaling pathways and promoting angiogenesis [33]. Moreover, as the permeability of new blood vessels increases, contrast agents can more easily enter the extracellular matrix of the tumor, hence resulting in a shorter RT [34]. HER2‐low breast cancer patients have relatively less angiogenesis, which affects the perfusion intensity of the contrast agent and RT.

This study revealed that the DVPC trend in HER2‐low tumors was initially positive and then negative, indicating that, during contrast agent infusion to the peak, the signal enhancement within the tumor was greater than that in the reference area; after reaching the peak, the contrast agent rapidly declined. After the peak was reached, the contrast agent signal within the tumor was notably weaker than that in the reference area. This change in wave direction may be due to heterogeneity within HER2‐low breast cancer tumors [35], resulting in differences in metabolism and neovascularization in different areas of the tumor.

We also explored the interaction between different parameters and found that the combination of Emax and RT consistently outperformed the individual feature with the higher AUC, sensitivity, and specificity in both groups. Moreover, the diagnostic ability of perfusion defect and homogeneity features was limited, but some studies also highlighted their potential diagnostic value. For example, HER2‐negative breast cancers, particularly the triple‐negative subtype, were more likely to exhibit perfusion defects due to limited neovascularization [11]. HER2‐overexpression had been shown to promote tumor angiogenesis by upregulating the VEGF signaling pathway, leading to the formation of dense microvascular networks and accompanied by the formation of perforating vessels showing the enhanced tumor perfusion on CEUS [8]. The PI3K‐AKT signaling pathway was a key regulator of tumor angiogenesis of HER2‐low breast cancers, and the aberrant activation could lead to the formation of immature, spatially heterogeneous vasculature and uneven perfusion, so the imaging findings may thus serve as an indirect predictor [8]. A related study found that CEUS‐derived perfusion characteristics (such as enhancement intensity, vascular distribution, and perfusion defect) were significantly associated with HER2 expression levels and could assist in the preoperative classification of breast cancer subtypes [33].

**FIGURE 13:**
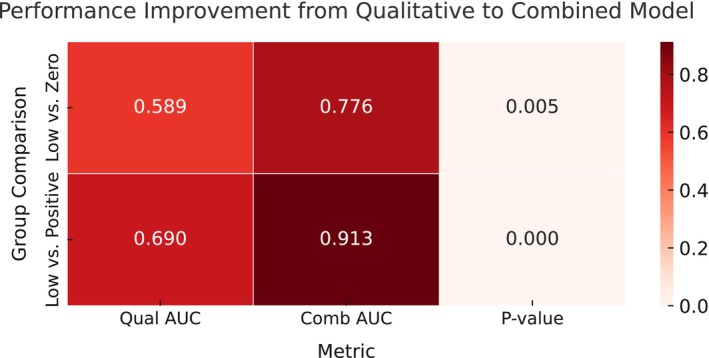
Comparison of model performance in HER2‐low breast cancer classification. Heatmap illustrates AUC improvement from the quantitative model (Emax + RT) to the combined model (quantitative + qualitative CEUS features) for distinguishing HER2‐low from HER2‐negative/positive subtypes. Significance was assessed using DeLong's test.

In our study, combined with the qualitative and quantitative ultrasound parameters were also analyzed for exploring the interaction in improving the diagnostic performance. We found there was excellent diagnostic performance based on perfusion defect, enhancement homogeneity, Emax, and RT in the HER2‐positive/low group (AUC = 0.913). In the HER2‐low/negative group, the AUC also could achieve 0.776. These findings further demonstrate that integrating qualitative and quantitative ultrasound features could enhance the diagnostic performances and hold promising clinical application potential, seen in Table 8, Figures 7, 8, and 13. Domestic and international scholars have conducted related research on the imaging diagnosis of HER‐2‐low breast cancer. For example, Whitney et al. [36] explored the application value of a DCE‐MR Radiomics Prediction Model for identifying HER2‐low‐expressing and HER2‐positive breast cancer and distinguished HER2‐low from HER2‐positive breast cancer on the basis of dynamic contrast‐enhanced MR, which achieved an AUC of 0.87 with an accuracy of 0.80, a sensitivity of 0.89, and a specificity of 0.82. Du et al. [14] used 2D ultrasound and radiomics to build a diagnostic model for HER‐2‐low, HER‐2‐negative, and HER‐2‐positive status in breast cancer, with an AUC of 0.840 for the HER‐2‐negative/low group and 0.81 for the HER‐2‐negative/positive group. Our study, which was based on multimodal ultrasound imaging (2D grayscale ultrasound, CDFI, SWE, and CEUS), predicted HER‐2‐low breast cancer. The former group achieved an AUC of 0.876 (95% CI = 0.780–0.953), with a sensitivity of 0.833, a specificity of 0.781, a PPV of 83.3%, an NPV of 78.1%, and an *F*1 score of 83.3%. The latter group achieved an AUC of 0.929 (95% CI = 0.855–0.978), with a sensitivity of 0.905, a specificity of 0.856, a PPV of 84.4%, and an NPV of 83.3%, indicating excellent performance. By applying SWE and CEUS imaging, especially some quantitative parameters, this study provided more abundant and objective information, such as tumor stiffness and blood perfusion, for the prediction of HER‐2‐low breast cancer. The predictive results of the model were validated with pathological findings via Kappa testing, with a Kappa value of 0.614 for the HER‐2‐negative/low group and 0.647 for the HER‐2‐positive/low group (*p* < 0.001), indicating good consistency. The Kappa coefficient was an indicator of the consistency between predicted outcomes and actual classifications, and a higher value indicated that the model maintains strong discriminative ability in the case of an imbalance in the sample size of the groups, thereby enhancing the clinical reliability of its predictions. In addition, DCA and calibration curves were also used to further evaluate the clinical utility and agreement between predicted and actual outcomes.

There were still some limitations in our study. First, the retrospective study may result in selection bias. Second, due to cost constraints, the number of cases undergoing CEUS was limited. Third, although HER2‐low breast cancer was a biologically heterogeneous group, we did not further stratify the population based on hormone receptor (HR) status (e.g., HR‐positive vs. triple‐negative) due to the limited sample size. Therefore, we plan to expand the number of cases using CEUS and perform the deep and subtype‐specific analysis of HER2‐low breast cancer in future multicenter studies.

## 5 Conclusion

This study provides imaging‐based evidence supporting the view that HER‐2‐low breast cancer is a distinct subtype. Compared with HER‐2‐negative breast cancer, HER‐2‐low cases were associated with low Ki‐67 expression, PR positivity, low enhancement levels, short RTs, and low Emax values. Compared with HER‐2‐positive tumors, patients with low Ki‐67 expression, ER and PR positivity, low Emax values, and an initial increasing trend followed by a decreasing DVPC were more likely to exhibit low HER‐2 expression. In conclusion, multimodal ultrasound imaging combined with clinicopathological information could effectively predict HER‐2‐low breast cancer.

## Author Contributions

**Feihang Dai:** writing – original draft, methodology, software. **Baoliang Guo:** conceptualization, methodology, data curation. **Xin Ai:** validation, project administration. **Dandan Liu:** conceptualization, data curation. **Longbin Dai:** supervision, investigation. **Chengzhi Zhang:** validation, formal analysis, writing – review and editing. **Xiaoping Leng:** conceptualization, methodology. **Tong Wu:** writing – review and editing, funding acquisition, validation, data curation.

## Ethics Statement

This study was performed in line with the principles of the Declaration of Helsinki. The Medical Ethics Committee of the Second Affiliated Hospital of Harbin Medical University approved this retrospective study (No. YJSKY 2024‐041).

## Consent

Informed consent was obtained from all individual participants included in the study.

## Conflicts of Interest

The authors declare no conflicts of interest.

## Data Availability

The datasets analyzed during the current study are available from the corresponding author on reasonable request.
